# Phytochemicals as Invaluable Sources of Potent Antimicrobial Agents to Combat Antibiotic Resistance

**DOI:** 10.3390/life13040948

**Published:** 2023-04-04

**Authors:** Ragi Jadimurthy, Swamy Jagadish, Siddaiah Chandra Nayak, Sumana Kumar, Chakrabhavi Dhananjaya Mohan, Kanchugarakoppal S. Rangappa

**Affiliations:** 1 Department of Studies in Molecular Biology, University of Mysore, Manasagangotri, Mysore 570006, India; jadimurthy.ragi@gmail.com (R.J.); jagadishsg@yahoo.com (S.J.); 2 Department of Studies in Biotechnology, University of Mysore, Manasagangotri, Mysore 570006, India; moonnayak@gmail.com; 3 Department of Microbiology, Faculty of Life Sciences, JSS Academy of Higher Education and Research, Mysore 570015, India; 4 Institution of Excellence, Vijnana Bhavan, University of Mysore, Manasagangotri, Mysore 570006, India

**Keywords:** antibacterial, antifungal, antiviral, natural compounds, phytocompounds

## Abstract

**Simple Summary:**

Many microorganisms develop resistance to drugs through different mechanisms, and this process is called antimicrobial resistance. It is highly essential to discover new antimicrobials to kill pathogenic microbes that have developed antimicrobial resistance. Natural sources, including plants, have been serving as a great source of medicinally important compounds for the past several decades. In this article, we have discussed the antimicrobial properties of plant-derived compounds against drug-resistant human pathogens, including bacteria, fungi, and viruses.

**Abstract:**

Plants have been used for therapeutic purposes against various human ailments for several centuries. Plant-derived natural compounds have been implemented in clinics against microbial diseases. Unfortunately, the emergence of antimicrobial resistance has significantly reduced the efficacy of existing standard antimicrobials. The World Health Organization (WHO) has declared antimicrobial resistance as one of the top 10 global public health threats facing humanity. Therefore, it is the need of the hour to discover new antimicrobial agents against drug-resistant pathogens. In the present article, we have discussed the importance of plant metabolites in the context of their medicinal applications and elaborated on their mechanism of antimicrobial action against human pathogens. The WHO has categorized some drug-resistant bacteria and fungi as critical and high priority based on the need to develope new drugs, and we have considered the plant metabolites that target these bacteria and fungi. We have also emphasized the role of phytochemicals that target deadly viruses such as COVID-19, Ebola, and dengue. Additionally, we have also elaborated on the synergetic effect of plant-derived compounds with standard antimicrobials against clinically important microbes. Overall, this article provides an overview of the importance of considering phytogenous compounds in the development of antimicrobial compounds as therapeutic agents against drug-resistant microbes.

## 1. Introduction

Antimicrobial agents are drugs that are used to prevent and treat infections caused by bacteria, fungi, viruses, and parasites. Thousands of small molecules and peptides were isolated from natural sources such as plants, bacteria, fungi, and marine invertebrates, and some have demonstrated significant antimicrobial activity in preclinical settings and clinics [1,2]. Therefore, they have been used as standard antimicrobial drugs against microbial infections. The period between 1940 to 1965 is considered the golden era of antibiotics as many new antibiotics were introduced to modern medicine which revolutionized the treatment of bacterial infections [3]. Unfortunately, the phenomenon of antimicrobial resistance is becoming one of the primary health concerns across the globe, in which the pathogens do not respond to existing antimicrobial agents, which complicates the treatment regimen and thereby increases the mortality rate [4]. There is a swift spread of pan-drug-resistant bacteria at an alarming rate. 

The World Health Organization (WHO) has declared that antimicrobial resistance is one of the top 10 global public health threats facing humanity. As per the antibiotic resistance threats report (2019) of the Centers for Disease Control and Prevention (CDC, the United States), the annual death rate due to antibiotic-resistant infection is over 35,000 people in the United States alone [5]. Misuse and overuse of antimicrobial agents are the prime reasons for the development of resistance by microbes, which pose a serious health concern to mankind [6,7]. Microorganisms develop resistance to antimicrobials in various ways and we have comprehensively discussed the underlying mechanisms that are involved in the development of antibiotic resistance in bacteria in our previous report [8]. Despite continuous efforts, a marked number of effective antimicrobials have not been discovered in the last three decades. Many pharmaceutical companies are involved in the development of drugs for the treatment of non-communicable metabolic disorders that are of significant economic interest. It is the need of the hour to focus on the discovery and development of novel antibiotics to treat deadly infections caused by antimicrobial-resistant organisms.

Mother Nature is serving as a treasure house of medicinally important compounds that can be used against various human ailments, including cancer, malaria, inflammatory diseases, and microbial infections [9,10,11,12]. We earlier demonstrated the pharmacological activities of various natural compounds in preclinical models of different diseases [13,14,15,16,17]. Extensive screening and research advancements in the previous century led to the discovery of thousands of bioactive secondary metabolites from medicinally important plants. Plants have been serving as a great source of bioactive compounds, which are being tested in preclinical disease models and clinical trials. Natural compounds, or their semi-synthetic derivatives obtained from plants, have contributed to the development of drugs against microbial diseases and various human ailments. For instance, artemisinin, a sesquiterpene lactone obtained from *Artemisia annua*, is used as a therapeutic agent for the treatment of malaria that is caused by *Plasmodium falciparum*. Various traditional medicine systems, folklore, codified systems of medicine, ethnopharmacology, ayurvedic classical texts, or zoopharmacognosy propose that some plants can be used against microbial infections. Some of the plant-derived metabolites have shown good antibacterial, antifungal, and antiviral activities in preclinical settings, and they can be considered potential candidates to be examined in clinical trials. In the present article, we have comprehensively discussed the mechanism of action of selected plant metabolites that have shown promising antimicrobial (antibacterial, antifungal, and antiviral) activity against clinically important human pathogens. Although some articles have been published in a similar line, many of them have not focused on the effect of plant-derived natural compounds on pathogenic microorganisms that are listed by the WHO as threats to mankind. We have attempted to provide a holistic view of the effect of selected natural compounds that have shown good growth-inhibitory activity toward clinically prominent microorganisms. We have also emphasized the synergistic effect of natural compounds with standard chemotherapeutic agents against human pathogens. 

## 2. Phytochemicals as a Source of Antimicrobial Compounds

### 2.1. Antibacterial Agents Derived from Plants

**Figure 1:**
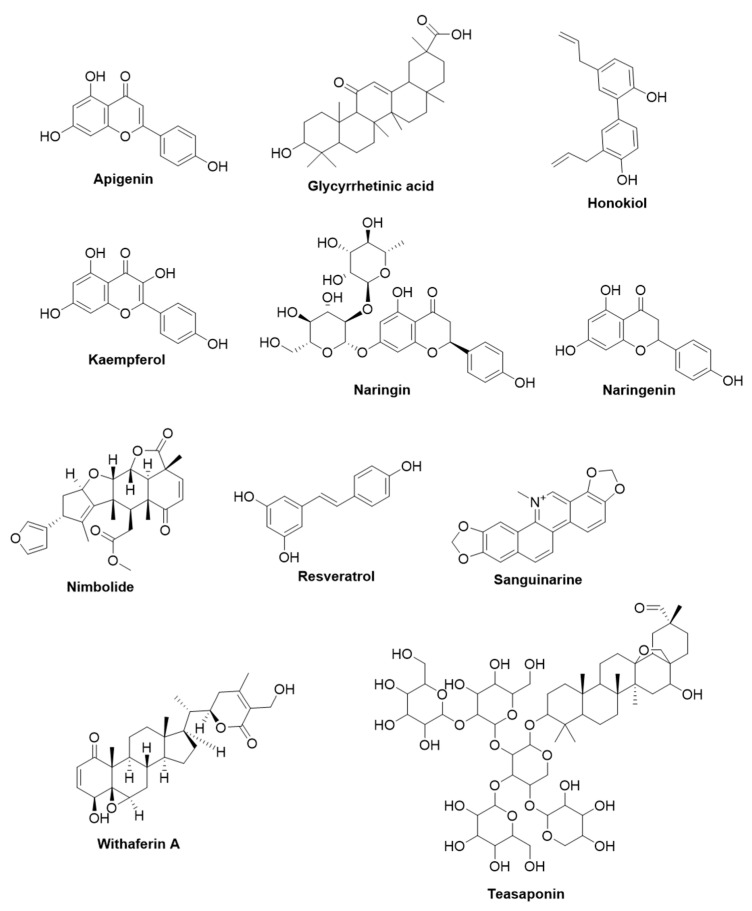
Chemical structure of phytocompounds with antibacterial activity.

Among microbial infections, bacterial infections pose a huge threat to human life across the globe. The WHO has categorized bacterial pathogens into critical, high, and medium priority depending on the need to develop new drugs against drug-resistant bacteria. The bacteria that are grouped under critical priority encompass carbapenem-resistant-*Acinetobacter baumannii* and -*Pseudomonas aeruginosa*, carbapenem-resistant, and third-generation cephalosporin-resistant Enterobacteriaceae. The bacteria that are categorized as high priority include vancomycin-resistant *Enterococcus faecium*, methicillin-resistant, vancomycin-resistant-*Staphylococcus aureus*, clarithromycin-resistant *Helicobacter pylori*, fluoroquinolone-resistant *Campylobacter* spp., fluoroquinolone-resistant *Salmonella* spp., and third-generation cephalosporin-resistant, fluoroquinolone-resistant-*Neisseria gonorrhoeae*. The medium priority list comprises penicillin-non-susceptible *Streptococcus pneumoniae*, ampicillin-resistant *Haemophilus influenzae*, and fluoroquinolone-resistant *Shigella* spp. [18]. Additionally, some of the bacteria included in these lists are also grouped as ESKAPE pathogens, which include *E. faecium*, *S. aureus*, *Klebsiella pneumoniae*, *A. baumannii*, *P. aeruginosa*, and *Enterobacter* spp., as they have adapted the escape mechanisms from the action of antibiotics. In the following section, we have discussed the mechanism of action of plant-derived natural compounds that target the abovementioned clinically important bacteria (Table 1). The structure of plant-derived compounds that are active against bacteria discussed in the text is given in Figure 1.

#### 2.1.1. Apigenin

Apigenin is a flavonoid found in various plants, including *Petroselinum crispum*, *Matricaria chamomilla*, *Apium graveolens*, *Basella rubra*, *Cynara scolymus*, *Origanum vulgare*, and *Portulaca oleracea* [19]. It was found to have antibacterial activity against *P. aeruginosa*, *K. pneumoniae*, *Salmonella typhimurium*, *Enterobacter aerogenes*, and *Proteus mirabilis*. Apigenin was found to inhibit *H. pylori*-derived D-Alanine:D-alanine ligase with a relatively lesser IC_50_ value (132.7 μM) than a positive control D-cycloserine (299 μM). Apigenin displayed a binding affinity towards *H. pylori*-derived D-Alanine:D-alanine ligase (k_D_ value: 22.3 μM), as demonstrated by surface plasmon resonance studies. In functional studies, apigenin displayed moderate antibacterial activity (MIC: 25 μg/mL) against *H. pylori* [20]. In a mass spectrometry-based assay to measure efflux pump inhibition, apigenin exerted efflux pump inhibition in *S. aureus* with an IC_50_ value of 38 µg/mL [21]. 

#### 2.1.2. 18-β-Glycyrrhetinic Acid

Glycyrrhizic acid is the primary saponin found in *Glycyrrhiza glabra* L., of the licorice family [22]. Glycyrrhizic acid and its derivatives are endowed with antibacterial, antitumor, antiviral, antibacterial, and anti-inflammatory activities [23]. Notably, glycyrrhizic acid is metabolically inactive and thus, it is metabolized to 18-β-glycyrrhetinic acid by the intestinal microflora upon consumption, leading to its absorption into the bloodstream. It was demonstrated that 18β-glycyrrhetinic acid-induced bactericidal activity against methicillin-resistant *Staphylococcus aureus* (MRSA) and its topical application significantly reduced staphylococcal skin and soft tissue infection in mice models. At the transcript level, pathogenicity-associated transcripts such as *saeR* (regulatory gene component of the global virulence regulatory system), *hla* (codes for α-toxin), *sbi* (gene essential for evasion of antibodies and complement), and *mecA* (gene associated with offering resistance to β-lactam antibiotics) were downregulated in 18-β-glycyrrhetinic acid-treated MRSA. A similar alteration in transcript abundance was obtained in bacterial mRNA isolated from the infected tissues of mice infected subcutaneously with MRSA [23].

#### 2.1.3. Honokiol

Honokiol [3′,5-di-(2-propenyl)-1,1′-biphenyl-2,2′-diol] is a bioactive neolignan found in the root bark of many species of the Magnoliaceae family, such as *Magnolia officinalis*, *Magnolia obovata*, and *Magnolia grandiflora* [24]. Honokiol has shown antibacterial potency against a wide range of bacteria from common oral pathogens to some of the ESKAPE organisms. Colistin is a last-line antibiotic that can be implemented in the treatment of multidrug-resistant (MDR) bacterial infections. Unfortunately, the emergence of *mcr-1* (a plasmid-mediated colistin resistance gene) is threatening the clinical use of colistin [25]. Guo and colleagues demonstrated that honokiol enhances the sensitivity of MCR-1-positive *Enterobacteriaceae* infections to polymyxin (polypeptide antibiotics) in vitro and in vivo. Molecular dynamics simulations showed that honokiol establishes hydrogen bonding and hydrophobic interactions with the active region of MCR-1 [26]. In another study, honokiol amphiphiles showed potent antibacterial activity against clinical isolates of MRSA (MIC: 0.5–2 µg/mL) with minimum cytotoxicity towards normal hepatocytes. These honokiol amphiphiles were found to disrupt biofilms and bacterial cell membranes, imparting bactericidal activity [27]. The major exopolysaccharide of the biofilm matrix of *S. aureus* is a chain of poly-N-acetyl-β-(1–6)-glucosamine, which is termed polysaccharide intercellular adhesin (PIA). Notably, the genes that code for enzymes essential for PIA synthesis are part of *ica* operon. Honokiol disintegrates existing biofilms of *S. aureus* by reducing the expression of biofilm-related genes (such as *sarA*, *cidA,* and *icaA*), decreasing the extracellular DNA release, and suppressing the expression of PIA [28]. In another study, honokiol was found to inhibit the secretion of α-hemolysin (an exotoxin released by *S. aureus* which selectively induces hemolysis of RBCs) and prevent α-hemolysin-induced hemolysis of rabbit RBCs. Honokiol protected mice from *S. aureus*-induced liver damage by suppressing the activation of the NLRP3 inflammasome and the expression of proinflammatory cytokines [29].

#### 2.1.4. Kaempferol

Kaempferol [3,5,7-trihydroxy-2-(4-hydroxyphenyl)-4H-1-benzopyran-4-one] is a flavonol abundantly present in tea (*Camellia sinensis*), broccoli (*Brassica oleracea*), apple (*Malus domestica*), and strawberry (*Fragaria x ananassa*) [30]. It has also been reported to be found in medicinal plants, including *Sophora japonica*, *Equisetum* spp., *Ginkgo biloba*, and *Euphorbia pekinensis* [19]. The antibacterial activity and mechanism of action of kaempferol have been demonstrated in various studies. Kaempferol inhibits the PriA helicase (an enzyme crucial for the restart of DNA replication and bacterial survival) activity of *S. aureus* [31] and displayed efflux pump inhibition in *S. aureus* with an IC_50_ value of 19 µg/mL [21]. In another study, kaempferol exhibited a synergistic effect with colistin against biofilm formation and growth of clinical isolates of colistin-resistant Gram-negative bacteria such as *P. aeruginosa*, *Escherichia coli*, *K. pneumoniae*, and *A. baumannii* [32]. The observed antibacterial activity was found to be mediated by disrupting the integrity of the cell membrane by kaempferol, which enables the increased interaction of colistin with the lipopolysaccharide of target bacteria. 

The pretreatment of kaempferol-3-O-glucorhamnoside (a derivative of kaempferol isolated from *Thesium chinense* Turcz) in mice challenged with *K. pneumoniae*, effectively downregulated the expression of important inflammatory mediators such as TNF-α, IL-6, IL-1β and PGE2 with parallel amelioration of lung edema. In addition, kaempferol-3-O-glucorhamnoside rescued RAW cells from the deleterious effects of *K. pneumoniae* infection [33]. Biofilm formation is one of the important factors responsible for offering resistance against antibacterial agents; therefore, the development of antibiofilm agents can increase the drug-sensitivity of bacteria. Attachment, maturation, and detachment are the three crucial phases in the development of the bacterial biofilm. Ming and colleagues identified that kaempferol suppresses the primary attachment phase of biofilm formation in *S. aureus* by decreasing the activity of sortase A and the expression of adhesion-related genes. *S. aureus* surface proteins (such as ClfA and ClfB) attach to the cell wall by sortase A and have a prominent role in the formation of biofilms [34].

#### 2.1.5. Naringin and Naringenin

Naringin (4′,5,7-trihydroxyflavanone-7-rhamnoglucoside) is a flavonoid glycoside that is excessively present in grapefruit and orange, whereas naringenin (5,7,4′-trihydroxyflavanone) is an aglycone form of naringin. Naringenin is effective against *Enterococcus faecalis*, a gram-positive bacterium present in the alimentary canal of humans and animals, which can cause life-threatening diseases in humans. Homology modeling and docking studies demonstrated that naringenin interacts with the active site of β-ketoacyl acyl carrier protein synthase Ⅲ, which is a key enzyme in the initiation of fatty acid synthesis in bacteria. The same study also demonstrated that naringenin displayed an antibacterial effect against *E. faecalis* (MIC: 256 µg/mL) [35]. The combination of naringenin with oxacillin and cloxacillin displayed synergistic antibacterial activity against MRSA [36]. The therapeutic application of naringenin is hampered due to its poor aqueous solubility and low bioavailability upon oral administration. Khan and coworkers prepared a naringenin-loaded, self-nanoemulsifying drug delivery system (NRG-SNEDDS) and examined the bioavailability upon oral administration. The total plasma concentration of NRG-SNEDDS was found to be significantly elevated compared to the naringenin control [37].

Several studies have demonstrated the antibacterial and antibiofilm effect of naringin in combination with standard antibiotics that are used in clinics. For instance, naringin potentiated the antibiofilm activity of ciprofloxacin and tetracycline on *P. aeruginosa* upon combinational treatment [38]. Naringin was reported to abrogate the biofilms of metallo-β-lactamases producing *Pseudomonas* species, which was evidenced by a remarkable reduction in the production of exopolysaccharides and alginate [39]. Zhou and colleagues reviewed the underlying antibacterial mechanisms of naringin and reported recently [40].

#### 2.1.6. Nimbolide 

Nimbolide (5,7,4′-trihydroxy-3′, 5′-diprenyl flavanone) is one of the vital limonoids present in the seeds, leaves, and flowers of *Azadirachta indica*, commonly known as neem [41]. It displayed bactericidal activity against *H. pylori*, a pathogen responsible for some diseases of the gastrointestinal tract, including peptic ulcers and gastric cancer. Merrell and colleagues demonstrated that neem oil extract possesses bactericidal activity [42]. Since *A. indica* has been reported to possess more than 300 phytochemicals, the same research group also demonstrated that nimbolide imparts bactericidal and antibiofilm activity against *H. pylori* [43]. In another study, nimbolide induced significant growth inhibitory activity against multi-drug-resistant (MDR) MRSA by damaging the membrane, lysis of bacterial cells, and disruption of biofilm [44].

#### 2.1.7. Resveratrol

Resveratrol (3,4′,5,-trihydroxystilbene) is a naturally occurring phytoalexin and is present in red wine, grapes, peanuts, berries, etc. [45,46]. It has exhibited antibacterial properties against a variety of organisms including *E. coli*, vancomycin-intermediate *Staphylococcus aureus* (VISA), *S. aureus, Campylobacter* species, and *Vibrio* species [47]. In an interesting study, the effect of resveratrol in combination with polymyxin B was examined against 50 MDR bacterial strains (26 strains of *K. pneumoniae* and 24 strains of *E. coli*), and among them, 44 strains were resistant to polymyxin B. Interestingly, resveratrol potentiated the antibacterial activity of polymyxin B against *K. pneumoniae* and *E. coli* [48]. Resveratrol has been reported to inhibit the electron transport chain and F_0_F_1_-ATPase, which contributes to the decline of ATP production and subsequent suppression of the growth of microorganisms [47]. It can also impart antibacterial activity by forming a copper-peroxide complex, upon which it interacts with DNA to form a DNA-resveratrol-copper ternary complex, which in turn ultimately results in the induction of DNA damage [49]. Another study indicated that resveratrol imparted growth-inhibitory activity against *E. coli* via inhibition of Z-ring formation through abrogation of FtsZ expression. FtsZ serves a pivotal role by assembling into a contractile ring (called the Z-ring) at the midcell site of the future septum during the division of bacteria [50]. The other mechanisms involved in the induction of antimicrobial effects by resveratrol against clinically important bacterial pathogens are comprehensively reviewed in the previous reports [47,51].

#### 2.1.8. Sanguinarine

Sanguinarine is a benzophenanthridine alkaloid obtained from the rhizomes of *Sanguinaria canadensis* L. (bloodroot), *Chelidonium majus* L. (Celandine), *Fumaria officinalis* L. (Fumitory), and *Bocconia frutescens* L. (Plume poppy). In *P. aeruginosa*, glucose enters the cell through the OprB and OprB2 porins and enters the periplasmic space, where it can directly enter the cytoplasm through an ABC transporter or it can be metabolized, which subsequently transported from the periplasm to the cytoplasm through the KguT (2-ketogluconate transporter). It has been shown that the *P. aeruginosa* mutant that lacks 2-ketogluconate transporter was relatively less pathogenic than wild-type *P. aeruginosa* [52]. Falchi and colleagues demonstrated that sanguinarine suppresses the 2-ketogluconate pathway of glucose utilization in *P. aeruginosa* by either targeting KguD or KguK [53]. In another report, sanguinarine induced an antibacterial effect against MRSA by triggering the release of membrane-bound cell wall autolytic enzymes, which eventually leads to bacterial lysis [54]. Interestingly, sanguinarine potentiated the antibacterial efficacy of standard clinically used antibiotics (such as ampicillin, oxacillin, norfloxacin, ciprofloxacin, and vancomycin) against MRSA [55].

#### 2.1.9. Withaferin A

Withaferin A (4-β,27-dihydroxy-1-oxo-5β,6β-epoxywitha-2,24-dienolide) is a natural steroidal lactone present in *Withania somnifera* and other members of the Solanaceae family, such as *Acnistus arborescens* [56]. Withaferin A displayed effective antibacterial activity against *P. aeruginosa* with a MIC and MBC of 60 µM and 80 µM, respectively. The effect was mediated by damaging the bacterial cell membrane. In addition, a significant reduction in the level of ROS and lipid peroxidation was reported upon withaferin A administration in *P. aeruginosa*-infected zebra fish larvae [57]. Metallo-β-lactamases are the antibiotic inactivating enzymes that contribute to the resistance against carbapenems. New Delhi metallo-β-lactamase (NDM-1) is contributing greatly to the emergence of antibiotic resistance among ESKAPE pathogens. In silico and in vitro screening studies revealed that withaferin A reduced the enzyme activity of New Delhi metallo-β-lactamase (IC_50_: 24.03 ± 2.9 μM). Withaferin A also displayed good synergistic activity with imipenem against clinical isolates of NDM-1-producing carbapenem-resistant *A. baumannii* with an FIC index value of 0.3125.

**Table 1:** List of phytochemicals that have demonstrated antibacterial activity against clinically important organisms.

Sl. No.	Phytocompound	Sources	Microorganisms Affected by the Title Compound and Dose	Mechanism of Action	Ref.
1	Allicin	*Allium sativum*, *Allium* spp.	*Streptococcus pneumoniae* (MIC: 64 µg/mL), *Streptococcus pyogenes* (MIC: 32 µg/mL)	ND	[58]
2	Conessine	*Holarrhena floribunda*, *Holarrhena antidysenterica*, *Funtumia elastica*	*P. aeruginosa* (MIC: 20 mg/L)	Inhibition of MexAB-OprM efflux pump	[59]
3	Thymol	*Thymus vulgaris*, *Thymus capitatus*	*K. pneumoniae* (MIC: 128 µg/mL)	Inhibition of biofilm formation	[60,61]
			*S. aureus* (MIC: 72 µg/mL)	Reversal of efflux pump action	
4	Carvacrol	*Origanum vulgare*	*S. aureus* (MIC: 256 µg/mL)	Reversal of efflux pump action	[61]
5	Eugenol	*Syzygium aromaticum*, *Eugenia caryophyllus*	*A. baumannii,* *Salmonella enteritidis*, *Campylobacter Jejuni,* *P. aeruginosa*, *E. coli*	ND	[62]
6	Berberine	*Berberis vulgaris*, *Berberis fremontii, Hydrastis Canadensis*	*H. pylori*	Increased the sensitivity of amoxicillin and tetracycline, and reduced the expression of *hefA* mRNA upon treatment with amoxicillin, tetracycline, and berberine	[63]
7	Curcumin I	*Curcuma longa*	*P. aeruginosa*	Damage to the bacterial membrane	[64]
			*H. pylori*	Inhibition of biofilm formation	
8	Quercetin	*Capparis spinosa*, *Polymnia fruticose, Ginkgo biloba*	*Salmonella enterica* serotype Typhimurium (MIC: 0.0072 µm/mL), *S. aureus* (MIC: 0.0068 µm/mL), *P. aeruginosa* (MIC: 0.0085 µm/mL)	Disruption of cell membrane integrity, thereby causing cell lysis	[65]
9	Epigallocatechin	*Camellia sinensis*	*S. aureus* (MIC: 62.5 µg/mL), *P. aeruginosa* (MIC: 125 µg/mL)	Increased the sensitivity of gentamycin against *S. aureus* and *P. aeruginosa*	[66]
10	Catechin	*Fructus Crataegi*	MRSA (MIC: 0.1 g/L)	Inhibition of biofilm formation via suppression of fibronectin-binding protein A and B (*fnbA* and *fnbB*)	[67]
11	Genistein	*Glycine max*	*Aeromonas hydrophila*	Disruption of QS, biofilm formation, and aerolysin production	[68]
12	Protocatechuic acid	*Scrophularia frutescens*	*Yersinia enterocolitica* (MIC: 2.5 mg/mL)	Cell membrane depolarization, reduction of intracellular pH and adenosine triphosphate (ATP), leakage of potassium ions	[69]
13	Gallic acid	*Vitis rotundifolia*	*P. aeruginosa* (MIC: 500 µg/mL), *S. aureus* (MIC: 1750 μg/mL), *Listeria monocytogenes* (MIC: 2000 μg/mL)	Membrane permeabilization, the release of intracellular potassium ions, disruption of the physicochemical surface properties of the cell	[70,71]
			*Shigella flexneri* (MIC: 2 mg/mL)	Inhibition of biofilm formation via regulation of *mdoH* gene expression and the OpgH protein	
14	Hydroquinone	*Vaccinium myrtillus*	*P. aeruginosa* (MIC: 7.81 µg/mL), *S. aureus* (MIC: 15.625 µg/mL)	Depolarization of the cell membrane potential, increase in cell permeability, and leakage of intracellular potassium ions	[72]
15	Osthole	*Cnidium monnieri, Angelica archangelica, Angelica pubescens*	*S. typhimurium* (MIC: 1.67 ± 0.58 µg/mL), *K. pneumoniae* (MIC: 3.33 ± 1.15 µg/mL), *A. baumannii* (MIC: 1.68 ± 0.58 µg/mL)	ND	[73]
16	Taxifolin	*Silybum marianum, Allium cepa, Pseudotsuga taxifolia, Pinus pinaster*	*E. faecalis* (MIC: 128 µg/mL), VREF (MIC: 512 µg/mL)	Based on docking data, taxifolin showed a good binding affinity for β-ketoacyl acyl carrier protein synthase III, which is an important enzyme for bacterial fatty acid biosynthesis	[74]
17	Piperine	*Piper nigrum*	MRSA (MIC: 100 µg/mL)	Liposomal formulation of piperine and gentamicin acts as an efflux pump inhibitor	[75]
			*S. aureus* (MIC: >16 µg/mL)	Piperine, in combination with ciprofloxacin, causes inhibition of efflux pump	
18	Sophoraflavanone B	*Desmodium caudatum*	MRSA (MIC: 15.6–31.25 µg/mL)	Disturbance of the cell membrane and leakage of cell contents	[76]
19	Farnesol	*Vachellia farnesiana*	*S. aureus* (MIC: 184 µg/mL), *L. monocytogenes* (MIC: 133 µg/mL)	ND	[77]
20	Rosthornin	*Rabdosia rosthornii*	*Propionibacterium acnes* (MIC: 3.17–25 µg/mL)	ND	[78]
21	Ellagic acid	*Rosa rugosa*	*H. pylori* (MIC: 5–30 mg/L)	ND	[79]
22	Chebulagic acid	*Terminalia chebula*	*A. baumannii*	ND	[80]
23.	Hexahydroxy diphenoyl ester vescalagin	*Lythrum salicaria*	*S. aureus* (MIC: 62 µg/mL), *P. mirabilis* (MIC: 62 µg/mL)	ND	[81]
24	Stigmasterol	*Neocarya macrophylla*	MRSA (MIC: 6.25–25 µg/mL), *Streptococcus faecalis* (MIC: 6.25–25 µg/mL)*, S. aureus* (MIC: 6.25–25 µg/mL)	Broad spectrum antibacterial activity	[82]
25	Chlorogenic acid	Fruits, vegetables, and graminaceous plants	*Streptococcus pneumoniae* (MIC: 20 µg/mL), *Salmonella typhimurium* (MIC: 20 µg/mL), *Shigella dysenteriae* (MIC: 10 µg/mL)	An increase in cell membrane permeability binds to bacterial DNA and thereby inhibits cellular functions	[83]
26	Thymoquinone	*Nigella sativa*	*S. flexneri* (MIC: 0.4 mg/mL)	Disruption of the cell membrane integrity	[84]
27	Guggulsterone	*Commiphora wightii* (Arn.) Bhandari	*E. coli* (MIC: 0.5 mg/mL), *K. pneumoniae* (MIC: 2 mg/mL), *P. aeruginosa* (MIC: >2 mg/mL), *Salmonella typhi* (MIC: >2 mg/mL), *E. faecalis* (MIC: 0.5 mg/mL), *S. aureus* (MIC: 2 mg/mL)	ND	[85]
28	Isoliquiritigenin	*Glycyrrhiza uralensis*	*Staphylococcus xylosus* (MIC: 80 µg/mL)	Downregulation of the IGPD gene	[86]
29	Celastrol	*Tripterygium* *Wilfordii*	*S. aureus* (MIC:1.25 µg/mL), *E. faecalis* (MIC: 1.25 µg/mL)	Disruption of DNA and protein synthesis	[87]
30	Cryptotanshinone	*Salvia miltiorrhiza* Bunge	*S. aureus* (MIC:12.5 µg/mL)	Dissipation of membrane Potential. Respiratory chain inhibition probably by targeting type II NADH:quinone dehydrogenase	[88]
31	Oridonin	*Rabdosia rubescens,* *Isodon rubescens*	MRSA (MIC: 64 µg/mL)	Permeability of cell membrane, disruption in protein and DNA metabolism	[89]
32	Magnolol	*Magnolia officinalis*	*S. aureus* (MIC: 16 ppm)	Based on simulation studies magnolol exhibited a high binding affinity for cell division Protein FtsZ	[90,91]
			MRSA (MIC: 10 µg/mL)	Repression of *mecA*, *mecI*, and upregulation of *mecR1*	
33	Hesperidin	citrus fruits, *Poncirus trifoliata*	*S. aureus* (MIC: 1 mg/mL), *Bacillus cereus* (MIC: 2 mg/mL), *E. coli* (MIC: >2 mg/mL) *P. aeruginosa* (MIC: 2 mg/mL)	ND	[92]
35	Evocarpine	*Evodiae fructus*	*Mycobacterium smegmatis* (MIC: 2–4 mg/mL), *Mycobacterium* *tuberculosis* (MIC: 5 mg/mL)	ND	[93]
36	Ursolic acid	*Malus domestica*	*K. pneumoniae* (MIC: 400 µg/mL), CRKP-1 (MIC: 800 µg/mL), CRKP-2 (MIC: 800 µg/mL), CRKP-8 (MIC: 800 µg/mL), CRKP-10 (MIC: 800 µg/mL)	Increase in membrane integrity, reduction in membrane potential, and intracellular ATP	[94]
37	Ferulic aid	All plants	*E. coli* (MIC: 100 µg/mL), *P. aeruginosa* (MIC: 100 µg/mL), *S. aureus* (MIC: 1100 µg/mL), *L. monocytogenes* (MIC: 1200 µg/mL)	Disruption of membrane integrity, cell surface hydrophobicity, and potassium ion leakage	[71]
38	Morusin	*Morus alba*	*S. aureus* (MIC:14.9 μmol/L)	Disruption of membrane integrity, Modulation of expression of phosphatidic acid biosynthesis-associated genes	[95]
39	Lonicerin	*Lonicera japonica*	*P. aeruginosa*	Inhibition of alginate secretion protein (AlgE) and inhibition of biofilm formation	[96]
40	Galangin	*Allium sativum*	VISA (MIC: 32 μg/mL)	Inhibition of murein hydrolase activity and growth of VISA strain-Mu50	[97]
41	Artemisinin	*Artemisia annua*	*S. aureus* (MIC: 0.09 mg/mL)	ND	[98]
42	Punicalagin	*Punica granatum*	*S. aureus* (MIC: 0.25 mg/mL)	Disruption of the cell membrane, leakage of potassium ions, Inhibition of biofilm formation	[99]
43	Aloe-emodin	*Cassia occidentalis*, *Aloe vera*, *Polygonum multiflorum* Thunb.	*S. aureus* (MIC: 32 μg/mL), MRSA (MIC: 16 μg/mL), *Staphylococcus epidermidis* (MIC: 4 μg/mL), *P. aeruginosa * (MIC: 256 μg/mL)	Transcriptional profile studies have revealed alterations of genes involved in sulfur metabolism, L-lysine, peptidoglycan biosynthesis, and biofilm formation	[100]
44	Skullcapflavone II	*Scutellaria* *baicalensis*	*M. smegmatis * (MIC_99_: 128 mg/L), *Mycobacterium aurum* (MIC_99_: 7.8 mg/L), *Mycobacterium bovis* (MIC_99_: 31.25 mg/L)	Efflux pump inhibition in *M. aurum* and *M. smegmatis*	[101]
45	Wogonin	*Agrimonia pilosa*	*P. aeruginosa*	Reduction of the quorum sensing-related genes. decreased production of virulence factors, inhibition of biofilm formation	[102]
46	Sulforaphane	*Brassica oleracea* and other cruciferous plants	*H. pylori* (MBC: 2.8–5.6 µg/mL)	Inhibition of bacterial urease	[103]
47	Arjunolic acid	*Syzygium guineense*, *Syzygium cordatum*	*Shigella sonnei* (MIC: 30 µg/spot)	ND	[104,105]
48	Terminolic acid	*Syzygium guineense*	*S. sonnei* (MIC: 50 µg/spot)	ND	[106]
49	Asiatic acid	*Centella asiatica*	*Clostridium difficile* (MIC: 10–20 μg/mL)	Disruption of membrane permeability, inhibition of cell motility	[107]
50	Cinnamic acid	*Cinnamomum cassia*	*M. tuberculosis* (MIC: 270 µM) *Neisseria gonorrhoeae* (MIC: 6.75 mM)	ND	[108,109]
51	Caffeic acid	Abundantly present in fruits and vegetables, such as olives, cinnamon, nutmeg, blueberries, apple, star anise	*S. aureus* (MIC: 256 µg/mL)	ND	[110]
52	Andrographolide	*Andrographis paniculata*	*Burkholderia pseudomallei* (MIC: 0.5 µg/mL)	Andrographolide-stabilized silver nanoparticle binding and charge neutralization at the membrane surface, and the production of Ag^+^ and ROS	[111,112]
			*P. aeruginosa*	Suppression of QS regulators *LasR* and *RhlR*, which control the expression of many genes in *P. aeruginosa*	
53	Diosgenin	*Rhizoma polgonati, Smilax china, Trigonella foenumgraecum*	*Porphyromonas gingivalis*, *Prevotella intermedia*	Inhibition of biofilm formation	[113]
54	Rhein	*Rheum palmatum, Reynoutria* *japonica* (Houtt.), *Fallopia multiflora*	*Cutibacterium acnes* (MIC: 6.25 µg/mL)	Inhibition of *C. acnes* NADH dehydrogenase-2 activity	[114,115]
			MRSA (MIC: 62.5–250 μg/mL)	Rhein in combination with oxacillin causes a reduction of *mecA/mecI/mecR1* and *blaZ/blaI/blaR1* gene expressions	
55	Riccardin C derivatives	*Reboulia hemisphaerica*	MRSA (MIC: 1 µg/mL), *E. faecalis* (MIC: 4 µg/mL), *P. aeruginosa* (MIC: >128 µg /mL), *Vibrio parahaemolyticus* (MIC: >128 µg/mL)	Disruption of membrane permeability and cell morphology, Alterations in intracellular Na^+^ and K^+^ concentrations, Mutation in *FabI* (an enoyl-acyl carrier protein reductase) in the *S. aureus* mutants	[116]
56	Artesunate	*Artemisia annua*	*M. tuberculosis* (MIC: 75 µg/mL)	ND	[117]
57	Betulinic acid	*Mikania cordata*	*P. aeruginosa* (MIC: 256 µg/mL), *S. aureus* (MIC: 256 µg /mL)	Increased production of a superoxide anion radical and malondialdehyde, elevated NAD^+^/NADH ratio, reduced glutathione, and DNA fragmentation	[118]
58	Sakuranetin	*Polymnia fruticosa*	*H. pylori* (MIC: 87.3 µM /mL)	Inhibition of β-hydroxy acyl-acyl carrier protein dehydratase	[119]
59	Protoanemonin	*Ranunculus bulbosus*	*S. aureus* (MIC: 31.25 µg/mL), *P. aeruginosa* (MIC: 62.5 µg/mL), *Serratia marcescens* (MIC: 15.625 µg/mL), *K. pneumoniae* (MIC: 31.25 µg /mL), *Providencia stuartii* (MIC: 15.625 µg/mL), *P. acnes* (MIC: 31.25 µg/mL), *Clostridium perfringens* (MIC: 62.5 µg/mL)	Broad spectrum antibacterial activity	[120]
60	Capsaicin	*Piper nigrum*, *Capsicum annuum*	*Streptococcus pyogenes* (MIC: 64–128 μg/mL)	Cell membrane damage, reduction of cell invasion and hemolytic activity, inhibition of biofilm formation	[121,122]
61	Thymoquinone	*Nigella sativa*	*P. aeruginosa* (MIC: 1.56 µg/mL), *S. aureus* (MIC: 3.125 µg/mL)	Depolarization of the membrane, production of ROS, and inhibition of biofilm formation	[84,123,124]
			*V. paraheamolyticus* (MIC: 32µg/mL), *Vibrio alginolyticus* (MIC: 256µg/mL), *Salmonella enterica* serovar Typhimurium (MIC: >512 µg/mL), *Staphylococcus epidermidis* (MIC: 8 µg /mL), *S. aureus* (MIC: 8 µg/mL)	Inhibition of biofilm formation	
			*S. flexneri* (MIC: 0.4 mg/mL)	Disruption of cell membrane integrity, inhibition of biofilm formation	
62	Piceatannol	Grapes, white tea, passion fruit, Japanese knotweed	*Streptococcus mutans,* *Streptococcus sanguinis*, *Streptococcus gordonii*	Inhibition of *Streptococcus* glucosyl transferase-GtfC	[125]
63	Curcumin	*Curcuma longa*	MRSA (MIC: 125–250 μg/mL), *E. faecalis,* *P. aeruginosa*	Membrane damage, inhibition of FtsZ proteins, inhibition of *mecA* gene transcription, reduced expression of PBP2α proteins	[64,126]
64	Reserpine	*Rauvolfia serpentina*	*S. aureus* (MIC:1200 µg/mL)	Inhibition of biofilm formation and virulence-regulatory proteins	[127,128]
			*M. tuberculosis*	ND	
65	Tomatidine	*Solanum lycopersicum*	*S. aureus*, *L. monocytogenes*, *Bacillus* species.	Inhibition of ATP synthase subunit C	[129]
66	Isoliquirtigenin	*Dalbergia odorifera, Glycyrrhiza* *uralensis*	*M. bovis* (MIC: 50 µg/mL), MRSA (MIC: 50–100 µg/mL)	Inhibition of FAS I and FAS II	[130]
67	2,2′,4-Trihydroxy chalcone	*Dalbergia odorifera*	*M. bovis* (MIC: 55 µg/mL)	Inhibition of FAS I and FAS II	[131]
68	Fisetin	*Rhus cotinus*	*M. bovis* (MIC: 63 µg/mL)	Inhibition of FAS II	[131]
69	Butein	*Rhus* *verniciflua*	*M. bovis* (MIC: 43 µg/mL)	Inhibition of FAS II	[131]
70	Coumarin	All plants	*S. typhimurium* (MIC: 2.5 mg/mL), *Enterobacter aerogenes* (MIC: 0.625 mg/mL)	ND	[132]
71	Plumbagin	*Plumbago rosea, Plumbago zeylanica*	*S. aureus* (MIC: 5 μg/mL), MRSA (MIC: 4–8 μg/mL)	Inhibition of DNA gyrase	[133]
72	Hibiscetin	*Hibiscus sabdariffa*	*K. pneumoniae* (MIC: 1024 μg/mL), *E. aerogenes* (MIC: 1024 μg/mL)	ND	[134]
73	Terchebulin	*Terminalia chebula*	*A. baumannii* (MIC: 500 μg/mL)	ND	[135]
74	Norwogonin	*Scutellaria baicalensis*	*A. baumannii* (MIC: 128 µg/mL)	ND	[135]

Abbreviations: CRKP: carbepenem-resistant-*Klebsiella pneumoniae*; FAS: fatty acid synthase; IGPD: imidazole glycerol phosphate dehydratase; MBC: minimum bactericidal concentration; MIC: minimum inhibitory concentration; ND: not determined; QS: quorum sensing.

### 2.2. Antifungal Agents Derived from Plants

Fungal infections in humans can be considered one of the low-key maladies in the antimicrobial research and healthcare sectors. Medical interventions (such as the use of catheters, intravascular or intracranial devices, neurosurgical procedures, the usage of contaminated devices, and the overuse of broad-spectrum antibiotics), treatment-associated immunosuppression (organ transplantations or stem cell transplantations), disease-associated immunosuppression (HIV infection), and co-infections (tuberculosis) are the risk factors abetting the fungal infections in humans [136]. COVID-19-associated fungal infections, such as mucormycosis, aspergillosis, and candidaemia, are recent examples of co-infections. Fungal infections are annually causing around 1.6 million deaths, which is higher than the deaths caused by tuberculosis (1.5 million deaths/year) [137,138]. The number of deaths related to fungal infections is increasing every year, which is posing a serious global health concern. Until recently, the WHO did not have any action plan or guidelines for fungal infections. On 25th October 2022, the WHO released its first-ever fungal priority pathogens list (FPPL) to direct and drive the research efforts towards life-threatening fungal pathogens, to accelerate international coordination, and to attract investments in research and development in therapeutics and diagnostics against the fungal infections, in addition to many other goals throughout the world. The WHO categorized fungal pathogens into three priority groups, i.e., critical, high, and medium priority, based on their antibiotic resistance status and their public health impact [139,140]. In the FPPL report, *Cryptococcus neoformans*, *Candida auris*, *Aspergillus fumigatus*, and *Candida albicans* are categorized under critical priority; *Nakaseomyces glabrata* (*Candida glabrata*), *Histoplasma* spp., *Eumycetoma* causative agents, *Mucorales. Fusarium* spp., *Candida tropicalis*, and *Candida parapsilosis* are kept under high priority; and *Scedosporium* spp., *Lomentospora prolificans*, *Coccidioides* spp., *Cryptococcus gattii*, *Pichia kudriavzeveii (Candida krusei)*, *Talaromyces marneffei*, *Pneumocystis jirovecii*, and *Paracoccidioides* spp. are listed under medium priority pathogens [139,140].

*Cryptococcus neoformans* stands as the top fungal pathogen as per multicriteria decision analysis (MCDA). It initially infects the lungs and later spreads to the central nervous system to cause lethal cryptococcal meningitis and cryptococcaemia with a mortality rate accounting for about 41% to 61% among the infected [141]. Unlike other fungal pathogens, this pathogen is not transferred from one person to another. Fluconazole, amphotericin B, and flucytosine are clinically approved drugs used for the treatment of *C. neoformans* infections. The mechanisms of antifungal resistance adapted by *C. neoformans* have not been precisely understood [139]. 

**Figure 2:**
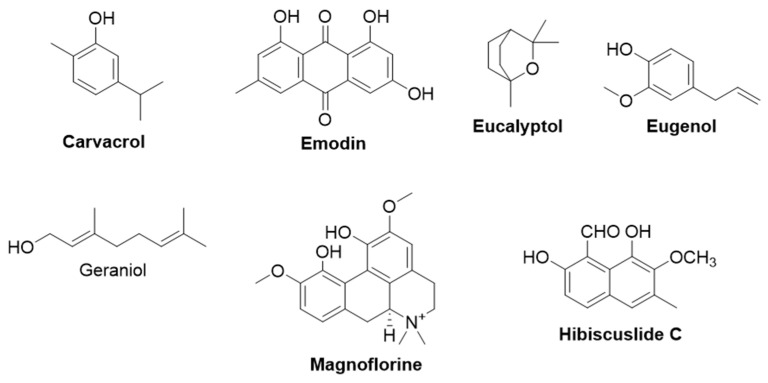
Chemical structure of phytocompounds with good antifungal activity.

*Candida auris* is the next ranked life-threatening fungal pathogen that causes invasive candidiasis, which affects the heart, central nervous system, eyes, bones, and internal organs [142]. Echinocandins, azoles, pyrimidines, and polyenes are the only four classes of antifungal agents used in clinics today. Importantly, 90% of *C. auris* isolates confer resistance to at least one class of antifungal agents, and 30% of the isolates display resistance against at least two classes of antifungal drugs [138]. In addition, other fungal pathogens provided in the priority list pose serious health consequences by developing resistance against antimycotics. An increasing number of fungal infections, the availability of a limited number of antifungal agents, and emerging antibiotic resistance demand the discovery of new antifungal agents. In the following section, we have discussed the effects and mechanisms of action of some of the plant-derived secondary metabolites with promising antifungal activity. (Table 2). The structure of plant-derived compounds that are active against fungi is given in Figure 2.

#### 2.2.1. Carvacrol 

Carvacrol (5-isopropyl-2-methylphenol) is a major constituent of essential oils obtained from the Lamiaceae family of plants, such as thyme and oregano [143]. Investigations carried out by Ahmed et al., (2011) showed the fungicidal activity of carvacrol against the various strains of fluconazole-sensitive and -resistant *candida* species, such as *C. albicans*, *C. tropicalis*, *C. parapsilosis*, *C. krusei*, *and C. glabrata* (mean MIC values of 75–90 mg/L for fluconazole-sensitive *candida* species and 75–100 mg/L for fluconazole-sensitive *candida* species) [144]. The study also suggested that the fungicidal activity of carvacrol could be due to interference with ergosterol biosynthesis and disruption of membrane integrity. Rao et al., showed that carvacrol disrupts Ca^2+^ and H^+^ homeostasis in *Saccharomyces cerevisiae*. Transcriptional profiling post-exposure to carvacrol showed a robust transcriptional response closely resembling that of calcium stress. It was speculated that the antifungal activity of carvacrol could be due to the induction of Ca^2+^ stress and inhibition of the TOR signaling pathway [144]. Chaillot et al., (2015) demonstrated that carvacrol disrupts the integrity of the endoplasmic reticulum, which in turn leads to endoplasmic reticulum stress and unfolded protein response in *C. albicans* [145]. In another study, carvacrol imparted cell death in *C. albicans* by increasing ROS levels, disrupting the mitochondrial membrane potential, causing DNA fragmentation and metacaspase activation, increasing cytosolic and mitochondrial Ca^2+^ levels, and activating calcineurin [144].

**Table 2:** List of phytochemicals that have demonstrated antifungal activity against clinically important organisms.

Sl. No.	Phytocompound	Sources	Microorganisms Affected by the Title Compound and Dose	Mechanism of Action	Ref.
1	Carvone	*Carum carvi*, *Anethum graveolens*, *Mentha spicata*	*C. albicans*	Inhibition of the transition from yeast form to filamentous form	[146]
2	Thymol	Present in the plants belong to genera such as *Thymus*, *Ocimum*, *Origanum*, *Satureja*, *Thymbra*, *Monarda*	*Candida* species (MIC: 100 µg/mL)	Inhibition of H^+^ ATPase	[147]
			*C. neoformans*	Interferes in intracellular Ca^2+^ homeostasis, reduction in ergosterol content through HOG-dependent pathway, reduction in protein glycosylation	
3	Menthol	*Mentha piperita*, *Mentha longifolia*, etc.	*Aspergillus niger* (MIC: 150 µg/mL), *Aspergillus fumigatus* (MIC: 150 µg/mL), *Aspergillus flavus* (MIC: 100 µg/mL), *Aspergillus ochraceus* (MIC: 100 µg/mL), *Alternaria alternate* (MIC: 450 µg/mL), *Botrytis cinerea* (MIC: 400 µg/mL), *Cladosporium* spp. (MIC: 125 µg/mL), *Penicillium citrinum* (MIC: 100 µg/mL), *Penicillium chrysogenum* (MIC: 300 µg/mL), *Fusarium oxysporum* (MIC: 200 µg/mL), and *Rhizopus oryzae* (MIC: 250 µg/mL)	Decreased the fungal growth dose-dependently	[148]
4	Cinnamaldehyde	*Cinnamomum* Cassia, *Cinnamomum burmannii*	*Geotrichum citri-aurantii*	Disruption of cell wall permeability and integrity	[149]
			*C. neoformans* var. grubii (MIC_90_: 0.683 mg/mL)	Damage to the cell wall, induction of cell gigantism	
5	Citronellal	*Cymbopogon citrates*	*C. albicans* (MIC: 1 mg/mL)	Disruption of membrane homeostasis, inhibition of yeast to hyphal transition and biofilm formation	[150]
6	Wogonin	*Scutellaria baicalensis* Georgi	*T. rubrum* (MIC_50_: 0.06 mM), *A. fumigatus* (MIC_50_: 0.23 mM)	Perturbance in cell wall synthesis,	[151]
			*Trichophyton mentagrophytes* (MIC_50_: 0.03 mM)	Perturbance in cell wall synthesis and generation of reactive oxygen species	
7	Gallic acid	*Punica granatum*	*T. rubrum* (MIC: 43.75 μg/mL)	Inhibition of ergosterol biosynthesis, reduction in sterol 14α-demethylase P450 (CYP51) and squalene epoxidase activity	[152]
			*T.mentagrophytes*, *Trichophyton violaceum*, *Trichophyton verrucosum*, *Trichophyton schoenleinii* (Mean MIC: 54.17–83.33 μg/mL), *C. albicans* (Mean MIC: 12.5 μg/mL)	ND	
8	α-pinene	Eucalyptus plants	*C. parapsilosis* (MFC: 128 μg/mL)	Inhibition of pseudo-hyphae and promoting a marked reduction in blastoconidia	[153]
9	β-Asarone	*Acorus calamus*	*A. niger*	Reduces ergosterol content in the plasma membrane	[154]
10	Quercetin	*Morus alba*, *Camellia sinensis*, *Allium fistulosum*, *Calamus scipionum*, *Centella asiatica*, *Lactuca sativa*	*C. albicans*	Programmed cell death through mitochondrial dysfunction	[155,156]
11	Osthole	*Cnidii Fructus*, *Cnidium monnieri*	*Microsporum canis* (MIC: 1.95 µg/mL)	Decrease in 1,3-β-D-glucan and chitin contents	[157]
12	Plagiochin E	*Marchantia polymorpha*	*C. albicans*	Induction of the metacaspase-dependent apoptotic pathway, inhibition of chitin biosynthesis	[158,159]
13	Riccardin D	*Dumortiera hirsute*	*C. albicans*	Down-regulation of hypha-specific genes, such as *ALS1*, *ALS3*, *ECE1*, *EFG1*, *HWP1* and *CDC35*, leading to retardation of hypha formation	[160,161]
			Azole-resistant *C. albicans* strains (MIC_80_: 16 µg/mL)	Interferes in sterol biosynthesis	
14	Silibinin	*Silybum marianum*	*C. albicans*	Inhibition of biofilm development, disruption of cell membrane	[162]
15	Chlorogenic acid	Present in a wide variety of fruits, vegetables, olive oil, wine, and coffee	*C. albicans*	Induction of apoptosis by mitochondrial depolarization, production of reactive oxygen species, DNA fragmentation, externalization of phosphatidyl serine.	[163,164]

Abbreviations: HOG: high-osmolarity glycerol response; MFC: minimum fungicidal concentration; ND: not determined.

#### 2.2.2. Emodin

Emodin (1,3,8-trihydroxy-6-methyl-anthraquinone) is a secondary metabolite produced by plants, such as *Senna alata*, *Rumex abyssinicus*, *Odontites serotina*, *Reynoutria japonica*, *polygonum* spp., and *Rheum palmatum* [165,166]. It possesses a therapeutic potential against many human ailments, such as hepatitis, cancer, arthritis, cholelithiasis, Alzheimer’s disease, ulcerative colitis, pancreatitis, asthma, and many bacterial and viral infections [167]. The antifungal activity of emodin against *C. albicans* (MIC: 12.5 μg/mL) was demonstrated [168]. Emodin showed antibiofilm activity and inhibition of hyphal formation in *C. albicans* cells [169]. Emodin also inhibited 50% total kinase activity of *C. albicans* at concentrations beginning from 0.5 µg/mL [169]. Additionally, emodin reduced the activity of CK2 (the most pleiotropic kinase in *C. albicans* cells) with an IC_50_ value of 2.7 μg/mL [169]. Molecular docking studies of emodin with CK2 showed that emodin binds to the ATP binding pocket of CK2 to impart its activity. In another study, emodin was found to reduce the activity of (1,3)-β-D-glucan synthase from *C. albicans* and increased cell wall damage [168]. (1,3)-β-D-glucan is the major polysaccharide found in the fungal cell wall and it is synthesized by (1,3)-β-D-glucan synthase. (1,3)-β-D-glucan synthase is regarded as the major drug target for the development of antifungal drugs, and echinocandins (known antifungal drugs) are known to target (1,3)-β-D-glucan synthase to impart antifungal activity. 

Ma et al., (2020) isolated aloe-emodin (1,8-dihydroxy 3-(hydroxymethyl)-9,10-anthracenedione), a compound with a close structural resemblance with emodin, from the root and rhizome of *Rheum palmatum* and the leaves of *Aloe vera* [170]. Aloe-emodin was demonstrated to show antifungal activity through antimicrobial photodynamic therapy against *C. albicans* cells [170]. Antimicrobial photodynamic therapy is a novel, promising approach against drug-resistant microorganisms in which a photosensitizer compound is incubated with a microorganism and an appropriate wavelength of light is irradiated to it. Upon irradiation, the photosensitizer is excited and undergoes molecular collision with surrounding oxygen molecules, and generates ROS (such as superoxide anions, hydroxyl radicals, singlet oxygen, etc.). These ROS cause damage to cellular envelopes (such as the cell wall and cell membrane), ultimately leading to cell death. Ma and colleagues demonstrated that aloe-emodin can be used as a photosensitizer in antimicrobial photodynamic therapy against drug-resistant *C. albicans* cells [170].

#### 2.2.3. Eucalyptol 

Eucalyptol [1,8-Cineole (1,3,3-trimethyl-2-oxabicyclo [2.2.2]acetate)] is a major component of essential oils extracted from plants of *Eucalyptus* species, such as *Eucalyptus smithii*, *Eucalyptus globules*, etc. It is also obtained from the essential oils of other plants, such as tea trees, mugwort, rosemary, etc. [171]. Eucalyptol showed antifungal activity against *C. albicans* and *C. glabrata* (MIC_90_ value: 800 μg/mL) by increasing ROS generation, G_1_/S phase arrest, elevating membrane permeability, and disrupting mitochondrial membrane potential [172]. Gene expression analysis revealed that genes essential for hyphal cell wall protein (HWP1), secreted aspartyl proteinase (SAP1), and cell surface adhesion (ALS1) are downregulated [172]. Mishra and colleagues synthesized the eucalyptol/β-cyclodextrin inclusion complex-loaded gellan/PVA nanofibers (EPNF) and studied their antibiofilm activity against *C. albicans* and *C. glabrata* cells [173]. EPNF inhibited approximately 70% of biofilm formation in the aforementioned fungal cells. A time-kill assay showed that the antifungal activity of EPNF was prolonged compared to eucalyptol alone [173]. Mączka and colleagues comprehensively discussed the possibility of the replacement of antibiotics with eucalyptol in their article [171].

#### 2.2.4. Eugenol

Eugenol (2-methoxy-4-[2-propenyl] phenol) belongs to the class of phenylpropanoids and is present in the essential oils obtained from Cinnamomum and clove [174]. Pereira et al., (2013) studied the antifungal activity of eugenol against *Trichophyton rubrum*, which is responsible for causing dermatophytosis [175]. Eugenol inhibited the growth of different strains of *T. rubrum* with MIC values ranging from 64–512 µg/mL. The inhibitory growth activity was found to be mediated by causing membrane abnormalities, which include short, twisted hyphae and a reduction in conidia formation [175]. In another study, eugenol was reported to impart antifungal activity against *C. albicans* by inhibiting the synthesis of ergosterol, inducing oxidative stress, promoting lipid peroxidation, and increasing membrane permeability [176]. Similarly, eugenol displayed antifungal activity against clinical isolates of *C. glabrata* (MIC value: 128 μg/mL) by the inhibition of biofilm formation [177]. In addition, eugenol also caused an increase in ROS generation, cell lysis, and ergosterol content in the plasma membrane and reduced the enzyme activities of catalase, phospholipase, and proteinase [177]. The gene expression analysis using qRT-PCR revealed that exposure of eugenol to C. *glabrata* differentially modulated the levels of ergosterol synthesis genes (*ERG2*, *ERG3*, *ERG4*, *ERG10*, *and ERG11*), sterol importer (*AUS1*), GPI-anchored cell wall protein (*KRE1*), 1,3-β-glucan synthase (*FKS1*), and multidrug transporter (*CDR1*). The expression of *AUS1*, *KRE1*, and *FKS1* was reduced, whereas *ERG2*, *ERG3*, *ERG10*, *ERG11*, and *CDR1* were increased upon eugenol treatment in *C. glabrata* [177]. The reduction in membrane potential and release of cytochrome c was also observed in *C. glabrata* cells treated with eugenol, which indicates the activation of apoptosis [177].

Similarly, eugenol was reported to have antifungal activity against *C. gattii* and *C. neoformans* with GMIC values of 200 and 187 mg/L, respectively. Eugenol altered cellular morphology, increased oxidative burst, and promoted lipid peroxidation in *C. gattii* and *C. neoformans* cells [178]. Eugenol, along with cinnamaldehyde, showed an additive effect and inhibited the growth of *candida* species such as *C. albicans*, *C. glabrata*, *and Candida lusitaniae* [179]. Many investigations reported the antibiofilm activity of eugenol against *Candida* species. Eugenol inhibited the single and mixed biofilms of fluconazole-resistant *C. albicans* [180]. El-Baz et al., (2021) performed a molecular docking analysis of eugenol against Als3, one of the adhesive proteins responsible for the adhesion of *Candida* cells to host or surfaces of medical devices, which subsequently results in biofilm formation [181]. Computational studies demonstrated that eugenol showed the highest binding capacity to Als3 compared to 1,8-cineole, 2-phenylthiolane, and cinnamaldehyde, implying that eugenol may interfere with the adhesion of *Candida* cells. Based on all of these investigations, eugenol can be investigated as a therapeutic agent against fungal infections in the clinical setting.

#### 2.2.5. Geraniol

Geraniol (3,7-dimethylocta-trans-2,6-dien-1-ol) is a monoterpene alcohol and a major constituent of essential oils extracted from wild bergamot, rose, lavender, palmarosa, etc. [182]. Geraniol is commercially used as a fragrance material in deodorants and cosmetic products. It is reported to have anticancer activity against murine leukemia, hepatoma, and melanoma cells. Miron et al., (2014) studied the antifungal activity of geraniol against many dermatophytes (*Trichophyton mentagrophytes*, *T. rubrum*, *Microsporum canis*, and *Microsporum gypseum*) and yeasts (*C. albicans*, *C. krusei*, *C. glabrata*, *C. tropicalis*, *C. parapsilosis*, *C. neoformans*, *Trichosporon asahii*) [183]. Geraniol demonstrated potent antifungal activity against *Microsporum* strains and other dermatophytes with GMIC values of 19.5 and 25.4 µg/mL, respectively [183]. It also displayed moderate antifungal activity against yeasts compared to dermatophytes. The investigation of the mechanism of action of the antifungal properties of geraniol against *T. asahii* revealed the ability of binding of geraniol to ergosterol and subsequent membrane destabilization [183]. Sharma et al., (2016) reported the antifungal activity of geraniol against three *Candida* species, such as *C. albicans*, *C. tropicalis*, and *C. glabrata* with MIC values of 130 µg/mL, 80 µg/mL, and 130 µg/mL respectively [184]. Geraniol did not show significant toxicity as evidenced by a hemolytic assay, compared to fluconazole and amphotericin B [184]. Geraniol was found to be involved in the inhibition of H^+^-ATPase and cell disruption of membrane integrity by interfering in ergosterol biosynthesis. Similarly, Pereira et al., (2015) also studied the antifungal activity of geraniol against *T. rubrum* (MIC value: 16–256 µg/mL) and reported that geraniol causes damage to the cell wall and cell membrane through the inhibition of ergosterol biosynthesis [185]. However, Leite et al.,(2015) studied the antifungal activity of geraniol against *C. albicans* and reported that geraniol neither interacts with ergosterol nor the cell wall, and they demonstrated the inhibition of pseudo-hyphae and chlamydoconidia formation by geraniol [186]. The pseudo-hyphae formation provides a survival benefit to the fungus by evading the host’s phagocytic system and acts as one of the contributing factors for the virulence of *Candida* species. Dalleau et al.,(2008) showed that geraniol possesses antibiofilm activity in *C. albicans*, which inhibited more than 80% of biofilm formation [187].

#### 2.2.6. Hibiscuslide C

Hibiscuslide C (1-formyl-2, 8-dihydroxy-7-methoxy-6-methylnaphthalene) is a phytochemical reported to be present in plants, such as *Hibiscus taiwanensis* and *Abutilon theophrasti* [188]. Hibiscuslide C showed antifungal activity against *C. albicans*, *C. parapsilosis*, *Trichosporon beigelii*, and *Malassezia furfur*, with MIC values of 5, 5–10, 10, and 5 µg/mL, respectively [188]. The mechanism of antifungal property of hibiscuslide C against *C. albicans* was found to be due to its involvement in membrane disruptive mechanisms, such as membrane depolarization and pore formation [188]. The same study also demonstrated that hibiscuslide C induces apoptosis in *C. albicans* via increased ROS generation, an increase in intracellular Ca^2+^, metacaspase activation, mitochondrial dysfunctions such as membrane depolarization, and the release of cytochrome c [189].

#### 2.2.7. Magnoflorine

Magnoflorine is a phytochemical present in medicinal plants, such as *Phellodendron amurense*, *Sinomenium acutum*, *Thalictrum isopyroides*, *Magnolia officinalis*, and *Berberis kansuensis*. It is reported to possess many pharmacological properties, such as antidiabetic, anti-inflammatory, immunomodulatory, antioxidant, and antifungal activities [190]. Magnoflorine displayed antifungal activity against various *Candida* strains, such as *C. albicans C. tropicalis*, *C. parapsilosis*, and *C. glabrata* [191]. Magnoflorine also presented alpha-glucosidase inhibitory activity and antibiofilm activity at a concentration of 150 μM. In another study, magnoflorine demonstrated antifungal activity against dermatophytes, such as *T. rubrum* and *T. mentagrophyte* (MIC: 62.5 μg/mL) [192], and inhibited conidia formation, abrogated hyphal growth, and altered mycelia morphology (deformed growth, cytoplasmic contraction, and surface peeling) in *T. rubrum* [192]. In addition, magnoflorine also caused cell membrane damage, nuclear content leakage, decreased ergosterol content, and reduced the activities of squalene epoxidase and CYP51 [192].

#### 2.2.8. Tea Saponin

Tea saponin is a phytochemical that belongs to an oleanane-type pentacyclic triterpene that is distributed in plants, such as *Camellia oleifera* and *Camellia sinensis* [193]. Tea saponin is present in the seed cake, which is obtained as the byproduct during the extraction of oil from tea or camellia seeds. Tea saponin is a natural surfactant used extensively in the food, chemical, pesticide, and cosmetic industries. Tea saponin is endowed with many pharmacological properties, such as antimicrobial, anti-inflammatory, antioxidant, and antiallergic properties [194]. Li et al., (2020) demonstrated the antifungal activity of tea saponin against different strains of *C. albicans*, on which it showed a moderate growth inhibition (MIC: 64 µg/mL) compared to fluconazole (MIC: 0.5-128 µg/mL) [195]. They also investigated the effect of tea saponin on the process of filamentation in *C. albicans.* The yeast to hyphal form transition is known as the filamentation process, which plays a critical role in the pathogenicity of *C. albicans* [195]. Li et al., (2020) found that tea saponin and fluconazole arrested the filamentation process in *C. albicans* until 12 h, at 64 µg/mL and 2 µg/mL concentrations, respectively, whereas the extensive filamentation process was observed at 9 h in the control and 16 µg/mL in tea saponin groups [195]. Biofilm formation is one of the unique mechanisms adapted by pathogens to acquire resistance against the host’s immune system and antimicrobial agents. Tea saponin inhibited 80% of biofilm formation at a concentration of 64 µg/mL in *C. albicans*. The same study also demonstrated that inhibition of filamentation and biofilm formation by tea saponin is due to a reduction in the level of cAMP in *C. albicans* [195]. The investigations by Yu et al., (2022) showed that tea saponin isolated from *Camellia oleifera* seed cake inhibited the growth of *C. albicans*, *S. cerevisiae*, and *Penicillium* with MIC values of 0.078, 0.156, and 0.156 mg/mL, respectively [196]. The antifungal activity of tea saponin is attributed to its involvement in cell membrane damage, a reduction in cell adhesion and aggregation, and antibiofilm activity in *C. albicans*. Transcriptomics analysis also revealed that hyphae- and biofilm-related genes, such as ALS3, ECE1, HWP1, EFG1, and UME6, are downregulated in the presence of tea saponin [196]. These studies suggest that tea saponin can be further studied and developed as a potential antifungal agent.

### 2.3. Antiviral Agents Derived from Plants

**Figure 3:**
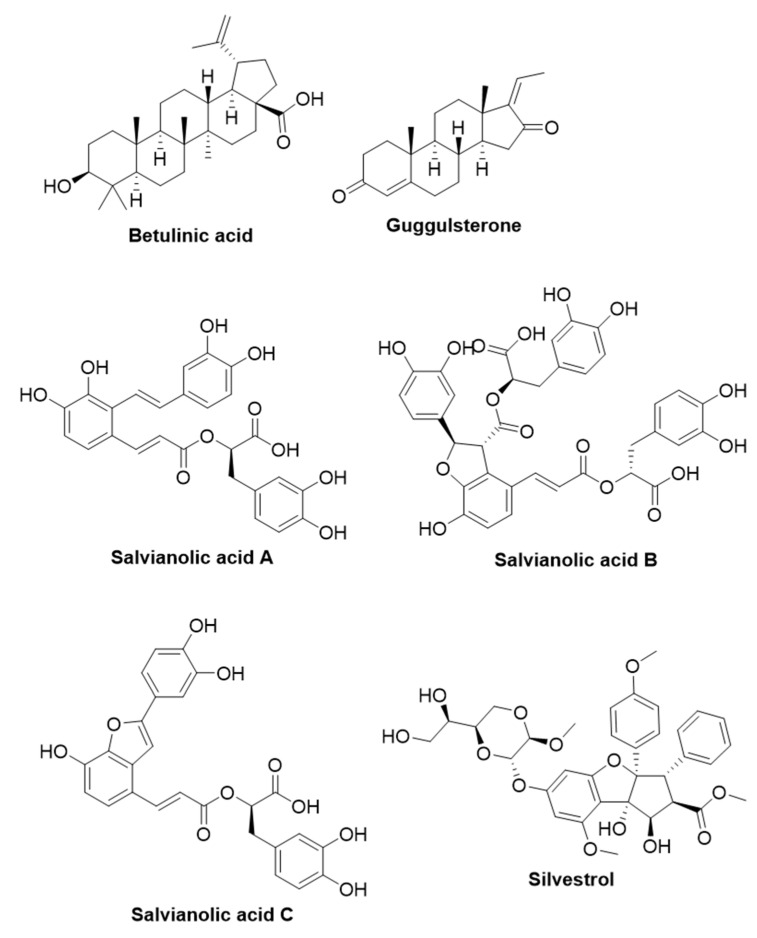
Chemical structure of phytocompounds with antiviral activity.

The knowledge of viral diseases in humans dates back to 1796, when Edward Jenner developed a vaccine against smallpox using the cowpox virus. In 1885, Louis Pasteur developed a vaccine against rabies [197]. In those days, the concept and existence of viruses were not known. In one of the early studies on the discovery of viruses, Dimitri Ivanovsky and Martinus Beijerinck identified an agent responsible for causing mosaic disease in tobacco plants. Studies by Ivanovsky and Beijerinck revealed that this infectious agent can pass through Chamberland ultrafilters. Beijerinck hypothesized that the ultrafilterable infectious agent is an infectious liquid that he called “contagium vivum fluidum” [198]. Subsequently, many ultrafilterable infectious agents (such as foot-and-mouth disease virus and myxoma virus in 1898, yellow fever virus in 1901, poliovirus in 1909, and many more) that are responsible for causing many diseases were identified. Ernst Ruska and Max Knol invented the electron microscope in 1933. Bodo von Borries, Helmut Ruska, and Ernst Ruska published the electron microscopic images of the mousepox virus and vaccinia virus in 1938, which laid the foundation for understanding the structure of viruses [199]. Thereafter, about 26 virus families that are known to infect humans have been identified, and about three to four new virus species infecting humans are being identified every year [200]. Human viruses implicate health impacts ranging from mild to life-threatening illnesses. As per the WHO statistics, the recent pandemic caused by the outbreak of the COVID-19 virus resulted in a mortality rate of 6,887,000 (as of 3 April 2023) [201]. The outbreak of Ebola viral infection in 2014 in West Africa resulted in a fatality rate of approximately 55% among the infected [202]. It is important to note that two-thirds of human pathogens are viruses. Viruses are highly adaptable biological entities that contribute to the emergence/reemergence of virus disease outbreaks. The study of the intricate “host-pathogen-environment” is crucial to understand these disease outbreaks [203]. It is important to discover novel antiviral compounds due to the high adaptability and rapid evolution of viral pathogens. In the following section, the antiviral efficacy of some of the selected plant-derived antiviral compounds has been discussed (Table 3). The structures of plant-derived compounds that are active against viruses are given as Figure 3.

#### 2.3.1. Betulinic Acid

Betulinic acid is a pentacyclic lupane-type triterpenoid widely present in different plant species [204]. It is generally isolated from the Birch tree (*Betula* sp., Betulaceae), which has well-known medicinal applications. Betulinic acid is also present in plants belonging to the genera *Ziziphus*, *Syzygium*, *Diospyros*, and *Paeonia* [205]. Many investigations revealed the antiviral potency of betulinic acid against different viruses. The antiviral function of betulinic acid against influenza A/PR/8 virus-infected A549 (human lung cancer) cells was examined [206]. Betulinic acid (50 µM) displayed good antiviral activity (98%) against the influenza A/PR/8/34 virus in A549 cells without significant cytotoxicity towards host A549 cell lines. Betulinic acid (10 mg/kg/dose for seven days) administration attenuated pulmonary pathological symptoms, including necrosis, number of inflammatory cells, and pulmonary edema in influenza A/PR/8/34 virus-infected C57BL/6 mice [206]. In general, the influenza A/PR/8/34 virus infection triggers the upsurge of proinflammatory cytokines (IFN-γ, IL-1β, and TNF-α) in the host, which leads to severe pulmonary inflammation. Interestingly, betulinic acid reduced the levels of IFN-γ in influenza A/PR/8/34 virus-infected C57BL/6 mice, indicating that betulinic acid may assist the recovery of infected mice by reducing severe pulmonary inflammation [206].

In another study, betulinic acid was reported to impart antiviral activity against the dengue virus type 2 (DENV-2). Betulinic acid (5 and 10 μM) reduced the viral titer 1.4 log_10_ fold in DENV-2-infected Huh7 (human liver cancer) cells. In vitro studies indicated that a 50% cytotoxic concentration (CC_50_), a 50% inhibitory concentration (IC_50_), and selectivity index values of betulinic acid against DENV-2 were found to be 28.24 µM, 0.9463 µM, and 29.843, respectively. The antiviral activity of betulinic acid was found to be due to its involvement in the inhibition of the post-entry stage of the DENV-2 replication cycle, viral RNA synthesis, and viral protein production [207].

#### 2.3.2. Guggulsterone

Guggulsterone is a phytosteroid present in the plant *Commiphora gileadensis* (L.), which is generally known as the “balsam of Mecca” [208]. *C. gileadensis* is known for its usage in the traditional Arabian medicinal system to treat urinary retention, jaundice, constipation, inflammatory disorders, and liver disorders [208]. This compound is also reported to be present in the plant Guggul tree (*Commiphora mukul*), and its medicinal values are well-documented in Ayurveda, a traditional Indian medicinal system [209]. Bouslama et al., (2019) studied the antiviral effect of methanolic extract of *C. gileadensis* leaves on two enveloped viruses (herpes simplex virus type 2 and respiratory syncytial virus type B) and two nonenveloped viruses (coxsackie virus B type 3 and adenovirus type 5). Methanolic extract of *C. gileadensis* leaves showed antiviral activity against enveloped viruses with an IC_50_ and a selectivity index of approximately 20 µg/mL and >10, respectively [208]. Subsequent bio-guided assays revealed that the leaf extract contains guggulsterone as the active compound. Chen et al., (2021) investigated the antiviral activity of guggulsterone against DENV and found that guggulsterone inhibited protein synthesis and RNA replication in DENV in a dose-dependent manner [210]. In vivo analysis in a ICR suckling mouse model demonstrated that guggulsterone stimulates the Nrf2-driven expression of heme oxygenase-1 to increase antiviral interferon response [210]. Hemeoxygenase-1 is a host antioxidant enzyme that breakdowns the heme ring into biliverdin. As per the previous reports, biliverdin inhibits DENV NS2B/NS3 protease activity, which is known to positively contribute to antiviral interferon response.

#### 2.3.3. Salvianolic Acids

Salvianolic acids are the class of phytochemicals present in *Salvia miltiorrhiza* (Danshen). The medicinal properties of *S. miltiorrhiza* have been recorded in traditional Chinese medicine and it has been known to promote blood circulation. *S. miltiorrhiza* contains about 10 different salvianolic acids and all of them have a common core chemical structure known as Danshensu [(R)-3-(3,4-Dihydroxyphenyl)-2-hydroxypropanoic acid] [211]. Out of these types, salvianolic acids A, B, and C are reported to have antiviral activity against SARS-CoV-2. Salvianolic acids demonstrate antiviral activity by binding to the SARS-CoV-2 spike (S) protein [212]. S protein is present on the surface of SARS-CoV-2 and interacts with angiotensin-converting enzyme 2 (ACE2), which is present in the host cells, to mediate the viral entry into the cells. Structurally, the S protein has two subunits, namely, S1 and S2, which are structurally distinct. S1 has a receptor binding domain that is involved in establishing an interaction with ACE2 on the host cell membrane. The binding of SARS-CoV-2 to ACE2 induces a conformational change in the S1 subunit, leading to the exposure of the S2′ cleavage site in the S2 subunit. Subsequently, the S2′ site is cleaved either by transmembrane serine protease 2 (TMPRSS2) present in the cell membrane (cell surface entry), or by cathepsins in the endosomes (endosomal entry pathway), which mark the two distinct SARS-CoV-2 entry pathways. The cleavage of the S2′ site in either pathway leads to shedding of the S1 subunit and the exposure of the fusion peptide (FP) domain in the S2 subunit, which subsequently leads to the insertion of the FP domain into the host cell membrane to facilitate membrane fusion. Additionally, the HR2 domain of the S2 subunit folds back and interacts with the HR1 domain, resulting in the formation of a six-helix bundle structure that brings the two membranes in close proximity and leads to the membrane pore formation through which the viral genome is injected into the host cell [213,214]. Yang et al., (2020) developed the pseudovirus model system using the SARS-Cov-2 S protein and studied the effect of salvianolic acid C (Sal-C) on the viral entry process in the host cells [215]. Sal-C inhibited the viral entry into the ACE2-expressing HEK293T and Vero-E6 cells with IC_50_ values of 3.85 and 0.47 μM, respectively. It was also shown using the plaque reduction assay that Sal-C reduced the number of plaques in the ongoing infection model (rather than the post-infection model), in which authentic SARS-CoV-2 was used [215]. The formation of the six-helix bundle core by the HR1 and HR2 domains of the S protein is a crucial event in the fusion of SARS-CoV-2 to the host cells. To understand the anti-SARS-CoV-2 activity of Sal-C, the authors synthesized HR1P and HR2P peptides, which contain the interacting regions of the HR1 and HR2 fusion core. Circular-dichroism spectroscopic analysis was performed to understand the biophysical change in the mixture of HR1P and HR2P peptides and HR1P or HR2P peptides alone. HR1P and HR2P peptides formed a HR1P/HR2P complex and showed a typical α-helical conformation of the six-helix bundle. Interestingly, the dose-dependent addition of Sal-C disrupted the characteristic α-helical conformation of the six-helix bundle in the HR1P and HR2P mixture. In addition, the dose-dependent treatment of Sal-C decreased the concentration of the six-helix bundle, as evidenced by the native-PAGE analysis [215]. These data concretely presented that the antiviral activity of Sal-C is due to its involvement in the disruption of the six-helix bundle conformation and thereby the abrogation of viral entry. The antiviral efficacy of salvianolic acids (Sal-A, Sal-B, and Sal-C) against SARS-COV-2 was studied in a pseudovirus system. For this, ACE2-overexpressing HEK293T cells were infected with 2019-nCoV spike pseudovirus. Sal-B displayed superior inhibitory activity over Sal-A and Sal-C towards the 2019-nCoV spike pseudovirus entry ratio, with an EC_50_ value of 6.22, 11.31, and 10.14 μM, respectively [212]. The mechanistic analysis also revealed that Sal-A, Sal-B, and Sal-C can suppress the entry of 2019-nCoV spike pseudovirus into ACE2-overexpressing HEK293T cells by interacting with the RBD of the spike protein and ACE2 [212].

#### 2.3.4. Silvestrol

Silvestrol (cyclopenta[b]benzofuran flavagline) is a secondary metabolite present in the species belonging to the *Aglaia* genus, and it is reported to have broad-spectrum antiviral activity against different viruses. Silvestrol imparts an antiviral function against the Ebola virus by inhibiting viral replication [216]. Silvestrol induces antitumor activity by binding to the eIF4A subunit of the eIF4F complex and thereby attenuates the translation of oncoproteins, such as c-MYC and PIM-1. The eIF4F complex contributes to the scanning of the 5′ untranslated region (UTR) of mRNA and the recognition of start codons by the ribosome to initiate translation. During Ebola infection, the virus delivers its RNA into the host cells, where viral transcription is initiated using it as a template. Additionally, the Ebola virus utilizes host cell machinery for the synthesis of viral proteins through a cap-dependent translation process. Biedenkopf and colleagues hypothesized that silvestrol can abrogate the viral translation as the viral mRNAs also contain 5′ cap and UTR regions (similar to eukaryotic cells) [216]. To determine the antiviral activity of silvestrol, Huh-7 cells were preincubated with silvestrol (10 nM), and then the cells were infected with the Ebola virus. The virus titers in the supernatant of infected cells were determined by performing TCID_50_ (50% tissue culture infectious dose) analysis by using Vero E6 cells, which demonstrated a significant reduction in the viral infection and the dose-dependent reduction in EBOV viral titer in the post-infection model. Silvestrol also inhibited the expression of viral proteins, such as VP40, NP, and GP proteins which could be due to the targeting of eIF4A by silvestrol [216]. In another study, 5′ UTRs of Ebola virus mRNAs were fused to a dual luciferase reporter plasmid (pFR_HCV_xb) containing the HSV-TK promoter and the firefly luciferase gene [217]. In this plasmid, hepatitis C virus internal ribosome entry site elements were placed downstream to the firefly luciferase gene, which helps in the translation of the Renilla luciferase gene, through the eIF4A-independent mechanism. The plasmid is transfected to HepG2 cells, and a dual-luciferase reporter assay was carried out. A decrease in the luciferase activity was observed for all Ebola viral 5’ UTR constructs upon treatment with silvestrol (10 nM), which emphasizes the significance of 5′ cap and UTR regions in the translation of Ebola viral proteins [217].

**Table 3:** List of phytochemicals that have demonstrated antiviral activity against human viruses.

Sl. No.	Phytocompound	Sources	Microorganisms Affected by the Title Compound and Dose	Mechanism of Action	Ref.
1	Berberine	*Berberis vulgaris*, *Berberis fremontii*, *Hydrastis Canadensis*	Chikungunya virus (EC_50_: 37.6–50.9 µM)	Reduction in viral RNA and protein synthesis	[218]
2	Baicalein	*Polygonatum sibiricum*, *Scutellaria baicalensis*	Japanese encephalitis virus (IC_50_: 14.28 µg/mL, CC_50_: 115.2 ± 0.2 µg/mL)	ND	[219,220]
3	Rosmarinic acid	*Salvia miltiorrhiza*	EV-A71 (CC_50_: 327.68 ± 14.43 µM, EC_50_: 31.57 ± 4.14–114 ± 4.10 µM, SI: 2.87–10.36)	Interferes with virus-host receptor interaction	[221]
4	Raoulic acid	*Raoulia australis*	HRV 2 (CC_50_: 201.78 µg/mL, IC_50_: <0.1 µg/mL, TI: 2017.8), HRV 3 (CC_50_: 201.78 µg/mL, IC_50_: 0.197 ± 0.11 µg/mL, TI: 1090.7), CV B3 (CC_50_: 65.86 µg/mL IC_50_: 0.337 ± 0.02, TI; 199.58), CV B4 (CC_50_: 65.86 IC_50_: 0.40 ± 0.05, TI: 164.65), EV 71 (CC_50_: 65.86 µg/mL, IC_50_: <0.1, TI: >658.6)	Broad spectrum antiviral activity	[222,223]
5	Tetra-*O*-galloyl-β-d-glucose (TGG)	*Galla chinensis*	SARS-CoV (CC_50_: 1.08 mM, EC_50_: 4.5 μM, SI: 240)	Interferes with viral entry into host cells	[224]
6	Saikosaponin B2	Bupleurum spp., Heteromorpha spp., Scrophularia scorodonia	HCoV-229E (IC50: 1.7 ± 0.1 mmol/L, CC_50_: 383.3 ± 0.2 μmol/L, SI: 221.9)	Interferes in virus absorption and penetration into host cells	[225]
7	Patentiflorin A	*Justica gendarussa*	HIV (IC_50_: 24–37 nM, CC_50_: 75 μM)	Inhibition of reverse transcriptase	[226]
8	Oligonol	*Litchi chinensis*	Influenza virus (H3N2)	Inhibition of the proliferation of the influenza virus by blocking ROS-dependent ERK phosphorylation	[227]
9	Punicalagin	*Punica granatum*	Influenza virus (H3N2)	Inhibition of agglutination of RBCs	[228]
10	3-hydroxy caruilignan C	*Swietenia macrophylla*	HCV (EC_50_: 10.5 ± 1.2 μM)	Inhibition of viral RNA and protein synthesis	[229]
11	Lycorine	*Lycoris radiate*, *Narcissus pseudonarcissus*	Zika virus (CC_50_: 4.29–21 μM, EC_50_: 0.22–0.39 μM, SI: 19.5–54)	Inhibition of viral RNA synthesis and protein synthesis, inhibition of viral RDRP activity	[230]
12	Quercetin	*Houttuynia cordata*	HSV-1 (CC_50_: 485.69 μg/mL, EC_50_: 52.9 μg/mL, SI: 9.18)	Inhibition of viral entry and NF-κB activation	[231]
			HSV-2 (CC_50_: 485.69 μg/mL, EC_50_: 70.01 μg/mL, SI: 6.94)	ND	
13	Shikonin	*Radix Lithospermi*	ADV-3	Inhibition of hexon protein expression	[232]
14	Naringenin	*Citrus paradisi*, *Citrus aurantium*, *Prunus cerasus*, *Solanum lycopersicum*, *Origanum vulgare*	HCV	Reduction in HCV secretion in infected cells	[233]
15	Ursolic acid	*Ocimum basilicum*	CV B1 (CC_50_: 100.5 mg/L, EC_50_: 0.4 ± 0.1 mg/L, SI: 251.3), EV 71 (CC_50_: 100.5 mg/L, EC_50_: 0.5 ± 0.2 mg/L, SI: 201)	Interferes in the viral replication phase	[234]
			HSV-1 (CC_50_: 100.5 mg/L, EC_50_: 6.6 ± 1.8 mg/L, SI: 15.2), ADV-8 (CC_50_: 100.5 mg/L, EC_50_: 4.2 ± 0.3 mg/L, SI: 23.8)	ND	
16	Myricetin	Abundant in fruit, vegetables, tea, berries	SARS-CoV-2	Inhibition of SARS-CoV-2 M^pro^ activity	[235]
17	Emetine	*Cephaelis* *ipecacuanha*	SARS-CoV-2 (CC_50_: 1603.8 nM, EC_50_: 0.147 nM, SI: 10,910.4)	Inhibition of SARS-CoV-2 mRNA/eIF4E interaction	[236]
18	Ladanein	*Marrubium Peregrinum*	HCV (EC_50_: 2.54 μmol/L, toxic dose 50 %: 98.04 μmol/L)	Interferes with virus entry into host cells	[237]
19	Samarangenin B	*Limonium sinense*	HSV-1 (IC_50_: 11.4 ± 0.9 μM)	Inhibition of HSV-1 α gene expression, inhibition of HSV-1 DNA synthesis, and structural protein expression	[238]
20	Pterocarnin A	*Pterocarya stenoptera*	HSV-2 (IC_50_: 5.4 ± 0.3 μM, CC_50_: 31.7 ± 1.6 μM, SI: 5.9)	Inhibition of virus attachment and penetration into host cells and inhibition of virus replication	[239]

Abbreviations: ADV: adenovirus; CV B: coxsackie virus B; ERK: extracellular signal-regulated kinase; EV: enterovirus; HCoV: human coronavirus; HCV: hepatitis C virus; HIV: human immunodeficiency virus; HRV: human rhinovirus; HSV: herpes simplex virus; ND: not determined; SARS-CoV-2: severe acute respiratory syndrome corona virus 2.

## 3. Synergistic Antimicrobial Effects of Plant Metabolites with Standard Antibiotics

The percentage of FDA-approved plant-derived antimicrobials is very insignificant (around 3%) compared to the abundance of plant metabolites [240]. Many traditional plant extract-based therapies involve the administration of a complex mixture of different phytochemicals that work in unison and may contribute to the synergistic effect to combat the growth of infectious microorganisms. Some researchers strongly believe that the synergistic potential of plant extract-based therapy might be a promising approach to address the rising antibiotic resistance [241]. In support of this, several plant-derived compounds have been demonstrated to potentiate the effect of antibiotics that are in clinical practice [242]. For instance, piperine, present in the *Piper nigrum* and *Piper longum*, inhibits bacterial efflux pumps to impart antibacterial activity. The nanoliposomes co-loaded with gentamicin and piperine showed synergistic antibacterial activity against MRSA and also reduced the MIC value of gentamicin about 32-fold [243]. Similarly, chanoclavine isolated from *Ipomoea muricata* also displayed bacterial efflux inhibition and presented a synergistic activity with tetracycline against MDR *E. coli* with a 16-fold reduction in the MIC of tetracycline [244]. Tomatidine, a secondary metabolite derived from the plants of tomato, potato, and eggplant, also demonstrated a synergistic effect with several aminoglycoside antibiotics against the MDR of *S. aureus* [245]. Thymol, a component of essential oil obtained from *Thymus vulgaris* and *Origanum vulgare*, displayed synergism with fluconazole against clinical isolates of Candida species such as *C. albicans*, *C. glabrate*, and *C. krusei* [246]. Epigallocatechin gallate (EGCG), a polyphenol present in tea leaves, showed synergistic antifungal activity with antimycotics such as miconazole, fluconazole, and amphotericin B against many Candida species [247]. These reports suggest that natural compounds obtained from plants can be used as potentiating agents of antimicrobial activity, and this fact can be considered in clinical trials.

## 4. Plant-Derived Drugs That Are in Clinical Practice for the Treatment of Human Ailments

Plants serve as an arsenal of secondary metabolites and their therapeutic applications against many infectious diseases are well-documented in ancient medical texts and paleobotanical findings at archeological sites [248,249]. Approximately 3% of natural products obtained from plants are approved by the FDA as antimicrobial agents, and an extensive portion of FDA-approved natural antimicrobial agents are obtained from microbes [240]. However, these reports may not reflect the true potential of phytochemicals as antimicrobial agents. According to the WHO, around 80% of the developing world is dependent on traditional medicine derived from medicinal plants [250]. In support of this, a huge number of drugs obtained from plants are in today’s clinical practice. Artemisinin, a phytochemical isolated from *Artemisia annua*, is widely used for the treatment of malaria, a life-threatening disease caused by *P. falciparum*. The discovery of artemisinin from plants is a breakthrough event in the research arena of plant-derived antimicrobial compounds. Apart from antimicrobials, many plant-derived compounds have also been developed as drugs against many human diseases. Approximately 25–28% of modern medicines are derived from plant sources [251]. For instance, galantamine, an isoquinoline alkaloid present in *Galanthus nivalis* and *Galanthus woronowii*, acts as an acetylcholinesterase inhibitor and is used in the treatment of Alzheimer’s disease [252,253]. Nitisinone is a chemical derivative of leptospermone, a phytochemical present in the plant *Callistemon citrinus*. Nitisinone is an inhibitor of 4-hydroxyphenylpyruvate dioxygenase and is used in the treatment of hereditary tyrosinemia type 1 [254]. Taxol, a blockbuster anticancer drug, was initially isolated from the bark of *Taxus brevifolia*, and was subsequently demonstrated to be produced by endophytes. Camptothecin is an approved drug that imparts an anticancer effect by inhibiting topoisomerase I and was initially identified to be produced by *Camptotheca acuminata*. Similarly, curcumin is a polyphenol present in the *Curcuma longa* and its medicinal applications are mentioned in ancient texts such as traditional Indian medicine and traditional Chinese medicine. It is considered a promising chemo-preventive agent against skin diseases such as psoriasis, vitiligo, and melanoma [255]. Curcumin is also reported to possess good antibacterial activity against different pathogenic microorganisms [256]. These examples provide a glimpse of the diverse therapeutic potential of phytochemicals. The logical drug repurposing approach also serves as an alternative approach for determining the antimicrobial activity of plant-derived drugs that are used against other diseases.

## 5. Conclusions and Future Perspectives

Antimicrobial resistance is one of the most serious health concerns across the globe, as many pathogens are rapidly developing resistance against existing antimicrobials. In the current scenario, there is no effective therapeutic agent with the potential to reverse antimicrobial resistance, and many leading laboratories are extensively working to discover new antimicrobials. Plant-based natural compounds are relatively less studied in the context of developing antimicrobial drugs. Natural compounds have been of great interest in the drug discovery process due to their structural diversity, chemical novelty, abundance, and bioactivity. Natural compounds have been isolated from various organisms, including bacteria, fungi, invertebrates, marine creatures, and plants. All of them have enormously contributed to the development of drugs against various human ailments. For instance, doxorubicin, bleomycins, epothilones, paclitaxel, camptothecin, podophyllotoxins, and vinca alkaloids are some of the well-known drugs derived either from bacteria, fungi, or plants [8,257,258]. In 2000, it was estimated that 57% of compounds that were undergoing clinical trials for cancer treatment were natural compounds [257]. It may be noted that many plant-derived metabolites have displayed antimicrobial activity against drug-resistant microorganisms, as discussed in the present article. A comprehensive investigation of the antimicrobial functions of plant metabolites needs to be carried out to explore their therapeutic potential. The plant metabolites can also be considered as scaffolds or template structures to chemically derivatize them to obtain compounds with improved antimicrobial efficacy. Additionally, the role of phytogenous compounds needs to be examined along with standard antibiotics to explore the possible synergistic effects. Overall, some plant metabolites have demonstrated good antimicrobial effects on clinically important microbes, and they could serve as future drugs against MDR pathogens.

## Data Availability

Not applicable.
